# Supplementary material for: Adenoma to Colorectal Cancer Estimated Transition Rates Stratified by BMI Categories—A Cross-Sectional Analysis of Asymptomatic Individuals from Screening Colonoscopy Program
Source: Cancers (Basel). 2021 Dec 23;14(1):62. doi: 10.3390/cancers14010062 (PMC8750540; doi:10.3390/cancers14010062)
Supplement: Supplementary file 1 [file cancers-14-00062-s001.zip › cancers-1517251-supplementary.pdf]

Supplementary Materials

Adenoma to Colorectal Cancer Estimated Transition Rates Stratified by BMI Categories—A Cross-Sectional Analysis of Asymptomatic Individuals from Screening Colonoscopy Program.

Piotr Spychalski, Jarek Kobiela, Paulina Wieszczy, Marek Bugajski, Jaroslaw Reguła and Michał F. Kaminski

Table S1. Transition rates with 95% confidence intervals.

nAA → AA	***eTR***	***95% CI***
Normal BMI	42.65%	40.97-44.39
Overwieght	41.81%	40.51-43.15
Obese	44.95%	43.10-46.86
AA → eCRC		
Normal BMI	9.02%	7.86-10.30
Overwieght	7.67%	6.82-8.59
Obese	8.39%	7.23-9.69
eCRC → aCRC		
Normal BMI	73.73%	62.75-86.08
Overwieght	69.90%	60.74-80.05
Obese	50.54%	40.84-61.85
